# AI Learning Self-Efficacy and Self-Perceived Digital Creative Functioning: A Conditional Indirect-Association Model

**DOI:** 10.3390/jintelligence14090213

**Published:** 2026-09-05

**Authors:** Yihui Liu, Jinming Sun, Weida Zhang

**Affiliations:** 1 School of Education, Jiangxi Normal University, Nanchang 330022, China; liu@jxutcm.edu.cn; 2 College of Humanities, Jiangxi University of Chinese Medicine, Nanchang 330004, China; 3 School of Education, Huaibei Normal University, Huaibei 235000, China

**Keywords:** generative artificial intelligence, AI learning self-efficacy, digital creative self-efficacy, self-perceived digital creative functioning, AI risk awareness, conditional process analysis, higher education

## Abstract

Generative artificial intelligence (GenAI) is increasingly embedded in higher education, but the association between students’ efficacy beliefs for AI-supported learning and their self-perceived digital creative functioning remains under-specified. This cross-sectional study tested a domain-specific efficacy account and examined a broad, study-specific AI learning risk-awareness measure as an exploratory boundary condition. Survey data from 920 Chinese higher education students were analyzed using confirmatory factor analysis and regression-based conditional process analysis with 5000 bootstrap resamples. The four focal measures showed a statistically distinguishable four-factor structure, although digital creative self-efficacy and self-perceived digital creative functioning remained conceptually close. AI learning self-efficacy was positively associated with self-perceived digital creative functioning, with a smaller statistical indirect association through digital creative self-efficacy. At mean risk awareness, the model-implied indirect point estimate was 0.177, compared with a direct association of 0.612. Conditional indirect point estimates decreased modestly from 0.196 to 0.157 as risk awareness increased. The conventional index of moderated mediation was negative and small and is interpreted here only as a statistical index of change in the conditional indirect association. These findings are consistent with a domain-specific efficacy account while indicating that the indirect component was meaningful but non-dominant. Single-wave self-reports preclude causal inference and do not constitute evidence of objectively rated creativity.

## 1. Introduction

Generative artificial intelligence (GenAI) refers here to computational systems that generate new content—such as text, images, audio, code, or multimodal material—in response to user inputs. In higher education, such systems now support knowledge search, ideation, feedback, revision, coding, translation, and multimodal production. This expansion has shifted the educational question from whether students can access AI to how they regulate it in learning and creative work (Chan, 2023; Crompton & Burke, 2023; Kasneci et al., 2023; Yan et al., 2024). Experimental and review evidence indicates that GenAI can broaden idea generation and improve some creative products, while convergence on similar suggestions may reduce collective diversity (Doshi & Hauser, 2024; Habib et al., 2024; Zhao et al., 2025). In the present survey context, examples included ChatGPT (GPT-4), Kimi, Tongyi, Doubao, and Wenxin Yiyan; the empirical interpretation therefore primarily concerns conversational and text-oriented GenAI use rather than all forms of generative systems.

Creativity involves producing ideas or products that are both novel and useful (Hennessey & Amabile, 2010; Runco & Jaeger, 2012). Digital creativity extends this definition to multimodal production, remixing, communication, and technology-mediated problem solving. The construct can be assessed through expert-rated products, behavioral tasks, portfolios, or self-reports, and these approaches do not provide interchangeable evidence (Hoffmann et al., 2016; Kaufman, 2019; Wu et al., 2026). Recent synthesis also identifies technological, individual, and contextual factors as distinct contributors to digital creativity (Wulan et al., 2025). The present study focuses on self-perceived digital creative functioning: students’ reports of their capability to generate, integrate, revise, and communicate digital outputs. This outcome is deliberately distinguished from objectively rated creative performance.

Technology-acceptance frameworks such as the Technology Acceptance Model (TAM) and the Unified Theory of Acceptance and Use of Technology (UTAUT) are highly relevant when the criterion is adoption intention or technology use, emphasizing constructs such as perceived usefulness, perceived ease of use, performance expectancy, social influence, and facilitating conditions (Davis, 1989; Venkatesh et al., 2003). The present question is different. The focal variables concern capability beliefs and self-perceived creative functioning among students already situated in GenAI-supported learning, rather than whether they intend to adopt a technology. Social cognitive theory was therefore selected as the primary framework because self-efficacy directly specifies task- and domain-specific capability judgments, persistence, self-regulation, and responses to difficulty (Bandura, 1986, 1997, 2001). TAM and UTAUT remain complementary frameworks for future research on adoption, continued use, or institutional conditions.

Social cognitive theory explains why students facing the same AI environment may report different patterns of engagement and perceived functioning. Efficacy beliefs shape exploration, persistence, goal regulation, and responses to difficulty (Bandura, 1986, 1997, 2001). AI learning self-efficacy concerns students’ judgments of their capability to use GenAI for learning tasks such as prompt formulation, output evaluation, and knowledge integration. Digital creative self-efficacy is narrower: it concerns capability judgments for carrying out creative digital tasks. A domain-specific account therefore predicts that broader efficacy beliefs about learning with AI being most closely associated with perceived digital creative functioning when they are accompanied by efficacy beliefs in digital creative action.

Recent research links AI self-efficacy to creativity-related outcomes directly or through AI literacy and critical thinking (S. Wang et al., 2023; Yang et al., 2026), but these models do not necessarily distinguish general AI-learning efficacy from creative-task efficacy and self-perceived enactment. Likewise, some empirical work operationalizes AI-related concern as anxiety (Y.-Y. Wang & Wang, 2022; Yang et al., 2026), whereas reviews of GenAI in education also document non-affective concerns about accuracy, overreliance, privacy, academic integrity, authorship, and bias (Yan et al., 2024; Zhai et al., 2024). Separating these adjacent constructs is necessary to clarify what a cross-sectional self-report model can and cannot establish.

The present study uses the content-based label AI learning risk awareness for a study-specific set of items concerning cognitive, ethical, privacy, evaluative, and academic risks. The label describes a broad risk-monitoring orientation rather than a validated replacement for AI anxiety. The content domains are heterogeneous, but they share a common response process: attending to possible adverse consequences or limitations of GenAI-supported learning. Risk awareness may coexist with efficacious use because experienced learners can engage with AI while monitoring its limitations. It may also qualify the association between creative self-efficacy and self-perceived functioning when verification demands, authorship uncertainty, or anticipated evaluation become salient. Given the study-specific operationalization and the absence of temporal evidence, this boundary condition is treated as exploratory.

Accordingly, the study has two aims. The first is to test whether AI learning self-efficacy is associated with self-perceived digital creative functioning indirectly through digital creative self-efficacy. The second is to explore whether AI learning risk awareness qualifies the latter association and the corresponding indirect estimate. The contribution lies in a more precise separation of general AI learning efficacy, creative-task efficacy, perceived creative functioning, and risk-aware monitoring. All paths are interpreted as cross-sectional associations rather than causal or developmental processes.

## 2. Literature Review and Hypothesis Development

### 2.1. AI Learning Self-Efficacy in GenAI-Supported Learning

Self-efficacy refers to beliefs about one’s capability to organize and execute actions required to attain desired outcomes (Bandura, 1997). In technology-mediated learning, these beliefs are associated with exploration, persistence, and strategic use of digital resources (Compeau & Higgins, 1995; Schunk & DiBenedetto, 2020). AI learning self-efficacy extends this logic to capability judgments concerning understanding AI functions, formulating prompts, evaluating generated outputs, integrating suggestions into one’s own learning products, and using AI consistently with academic norms (Long & Magerko, 2020; Y.-Y. Wang & Chuang, 2024). Self-efficacy is used here in Bandura’s capability-judgment sense; the manuscript avoids treating the broader everyday term confidence as an interchangeable construct.

GenAI interaction requires iterative dialogue, probabilistic evaluation, and ethical judgment. Students with stronger AI learning self-efficacy may therefore be more willing to test prompts, compare alternatives, diagnose errors, and revise AI-supported work. These behaviors do not establish creative performance, but they create conditions under which students may perceive themselves as more capable of digital creative functioning (Chan & Hu, 2023; Kasneci et al., 2023; S. Wang et al., 2023).

**H1.**  *AI learning self-efficacy is positively associated with self-perceived digital creative functioning.*

### 2.2. Digital Creative Self-Efficacy as a Domain-Specific Bridge

Creative self-efficacy is the belief that one can produce creative outcomes (Tierney & Farmer, 2002). Meta-analytic evidence shows a positive association with multiple creativity indicators, although the magnitude varies by measure and context (Haase et al., 2018). Digital creative self-efficacy contextualizes this belief for digitally mediated tasks: generating original ideas, developing digital solutions, combining modalities, and persisting through open-ended production challenges.

The proposed first link follows the principle of efficacy specificity. General capability beliefs can become task-specific through mastery experience, vicarious learning, feedback, and repeated engagement (Bandura, 1997, 2006). Students who judge themselves capable of learning with GenAI may be more willing to experiment with prompts, evaluate alternatives, and refine digital outputs. Such experiences are expected to be associated with stronger efficacy beliefs in the narrower domain of digital creative work.

Digital creative self-efficacy is, in turn, expected to be associated with self-perceived digital creative functioning. Efficacy beliefs can support persistence in ambiguous tasks, tolerance of uncertainty, exploration of alternative representations, and iterative revision (Beghetto, 2006; Gong et al., 2009; Tierney & Farmer, 2011). Because both variables are self-reported and use capability-oriented language, this hypothesis concerns related but conceptually ordered perceptions; it does not equate perceived functioning with externally evaluated creativity.

**H2.**  *AI learning self-efficacy is positively associated with digital creative self-efficacy.*

**H3.**  *Digital creative self-efficacy is positively associated with self-perceived digital creative functioning.*

**H4.**  *AI learning self-efficacy has a positive statistical indirect association with self-perceived digital creative functioning through digital creative self-efficacy.*

### 2.3. Distinguishing Digital Creative Self-Efficacy from Self-Perceived Digital Creative Functioning

Digital creative self-efficacy and self-perceived digital creative functioning occupy adjacent positions in the proposed model, but they are not intended to represent the same judgment. Consistent with social cognitive theory, self-efficacy is a prospective, task-specific judgment about whether one can organize and execute actions successfully, particularly when goals are demanding or obstacles arise (Bandura, 1997, 2006). Self-perceived digital creative functioning is broader and more enactment-oriented: it concerns students’ self-appraisals of generating, integrating, revising, expressing, and communicating digital outputs across their current activity. Conceptually, the former is a belief about capability for action, whereas the latter is a self-perception of enacted creative functions.

This distinction is nevertheless narrower than the distinction between self-efficacy and objectively rated creative performance because both measures are assessed through self-report, and several functioning items use capability-oriented wording. We therefore treat the two measures as indexing adjacent and only cautiously distinguishable self-perceptions rather than fully independent constructs. Throughout the manuscript, the outcome is labeled self-perceived digital creative functioning, and claims about actual creative behavior, product novelty, or product quality are avoided (Kaufman, 2019; Wu et al., 2026). The empirical evidence for statistical separation is presented in the measurement section rather than used as a substitute for this conceptual argument.

### 2.4. AI Learning Risk Awareness as a Boundary Condition

AI learning risk awareness is operationalized as a broad, study-specific monitoring orientation toward possible reliability, cognitive, ethical, privacy, evaluative, and academic consequences of GenAI-supported learning. The items cover inaccurate outputs, overreliance, originality, academic integrity, bias, verification, learning pressure, social misunderstanding, privacy, and passive learning. These domains are heterogeneous and are not assumed to be psychologically interchangeable. They are grouped here because they share a common referent: attending to possible adverse consequences or limitations of GenAI use in learning (UNESCO, 2023; Yan et al., 2024; Zhai et al., 2024). Accordingly, the score is used as a parsimonious empirical summary of broad risk monitoring in this sample. The observed one-factor internal structure is evidence of covariation among these indicators in this dataset, not proof that AI-related risks form a universal unidimensional psychological trait.

Competing patterns are theoretically plausible. Risk-aware learners may engage more deliberately, verify outputs, and preserve authorship, which could coexist with stronger efficacy and functioning. Conversely, sustained verification, uncertainty about originality, or anticipated evaluation may add cognitive or evaluative burden and modestly weaken the association between creative self-efficacy and perceived enactment. Because the existing theory and evidence do not uniquely privilege one directional prediction, and because the item set is study-specific and cross-sectional, the moderation is genuinely exploratory rather than a directional hypothesis disguised as a research question.

**RQ1.**  *Does AI learning risk awareness qualify the association between digital creative self-efficacy and self-perceived digital creative functioning?*

**RQ2.**  *Does the statistical indirect association between AI learning self-efficacy and self-perceived digital creative functioning through digital creative self-efficacy vary across levels of AI learning risk awareness?*

## 3. Materials and Methods

### 3.1. Research Design

This study used a quantitative, cross-sectional survey design to examine associations among AI learning self-efficacy, digital creative self-efficacy, self-perceived digital creative functioning, and AI learning risk awareness. All focal variables were measured in a single questionnaire administration. The design can evaluate covariance patterns and the internal structure of the proposed measures, but it cannot establish temporal precedence, developmental sequence, or causality.

The analytic strategy separated measurement evaluation from observed-score association testing. Confirmatory factor analysis assessed whether the four focal measures could be statistically differentiated within the proposed four-factor structure. Regression-based conditional process analysis then estimated the statistical indirect association through digital creative self-efficacy and explored whether risk awareness qualified the second-stage association. The first four hypotheses were theory-directed association hypotheses; moderation and conditional indirect variation were treated as exploratory research questions.

Although the multi-item measures were represented as latent factors in the CFA, the focal associations were estimated from composite scores. CFA was used as measurement-diagnostic evidence for the internal structure of the multi-item measures; the subsequent regressions do not inherit latent-variable correction for measurement error. The inferential target of the focal analysis is therefore the conditional association among observed scale scores. Regression-based conditional process estimation permits transparent bootstrap probing of the second-stage interaction and its conditional indirect association at the observed-score level (Hayes, 2018; Preacher et al., 2007). A latent-variable interaction SEM would explicitly model measurement error but would require a different estimator and additional assumptions. We therefore report the structural results explicitly as observed-score associations and identify latent-variable SEM as an important robustness extension rather than treating composite-score regression as equivalent to SEM.

### 3.2. Participants

Participants were higher education students in China. A total of 966 returned questionnaires entered screening. After incomplete responses, patterned responses, and responses with implausibly short completion times were excluded, 920 usable questionnaires remained, representing a usable-response retention rate of 95.24%. This percentage describes retention among returned questionnaires and should not be interpreted as a population response rate. The final sample was large relative to the 44-item, four-factor measurement model and the observed-score conditional process model, although sampling composition remains relevant to generalizability. Demographic characteristics are summarized in Table 1.

Table 1 shows that the sample was concentrated among first-year students (59.0%), students enrolled at regular undergraduate universities (82.0%), and students in STEM disciplines (56.6%). These composition features are relevant to the scope of inference. Beyond demographic composition, participants also differed in their prior exposure to digital learning and GenAI. Table 2 therefore reports weekly digital-learning time, GenAI-use duration and frequency, prior digital-creation/project experience, and self-rated digital skill.

The background profile in Table 2 indicates meaningful variation in technology exposure: 51.6% of participants reported more than one year of GenAI use, while 33.8% reported using GenAI for learning almost every day. These background characteristics provide contextual information for interpreting the focal self-reports and are treated descriptively in the primary analysis rather than as prespecified causal covariates.

### 3.3. Procedure and Ethical Considerations

Data were collected through an online questionnaire after institutional ethics approval. Demographic and background questions preceded the focal measures. The questionnaire included AI learning self-efficacy, digital creative self-efficacy, self-perceived digital creative functioning, AI learning risk awareness, and additional digital-learning background variables. The present article reports the four constructs used in the stated model; descriptive variables are summarized in Table 1 and Table 2.

Participation was voluntary and anonymous. Before beginning the questionnaire, students were informed of the study purpose, academic use of the data, confidentiality protections, their right to discontinue, and the absence of personally identifying information in the analytic dataset. Informed consent was obtained from all participants.

### 3.4. Measures

All focal items used a five-point Likert response format ranging from 1 (strongly disagree) to 5 (strongly agree). Higher composite scores indicated higher levels of the relevant operationalization. Appendix A provides the complete wording so that readers can inspect item content directly. The item sets were developed by mapping construct definitions and content domains in the cited studies onto the present GenAI and digital-learning context. They are therefore treated as study-specific contextualized operationalizations rather than exact reproductions of established instruments or newly validated general-purpose scales. The present article evaluates internal consistency and internal structure in this sample only; it does not infer content, criterion, predictive, or cross-cultural validity from high reliability or CFA fit. The available study records do not document a separate formal expert-panel review, pilot-testing study, or translation/back-translation validation protocol. We therefore do not claim that those procedures were completed and do not present these item sets as validated scales. This absence is treated as a measurement limitation, not merely a reporting omission. Independent content validation, piloting, translation-equivalence assessment where relevant, and cross-sample validation are required before broader scale use. Throughout the manuscript, self-efficacy refers to capability judgments in the social-cognitive sense; the word confidence is retained only when describing or reproducing item wording that explicitly uses that term.

#### 3.4.1. AI Learning Self-Efficacy

AI learning self-efficacy was represented by 10 study-specific items informed by AI self-efficacy and AI literacy research (Long & Magerko, 2020; Y.-Y. Wang & Chuang, 2024). Wang and Chuang’s original AI self-efficacy instrument is broader and multidimensional; the present items focus specifically on learning with GenAI. They cover capability judgments about learning new tools, prompt formulation, output evaluation, integration of generated content, organization of learning materials, problem solving, comparison of responses, independent learning, appropriate academic use, and integration with personal understanding.

#### 3.4.2. Digital Creative Self-Efficacy

Digital creative self-efficacy was represented by 10 contextualized items informed by creative self-efficacy research (Beghetto, 2006; Tierney & Farmer, 2002). The items use explicit confidence wording to operationalize self-efficacy judgments about generating original ideas, developing digital solutions, transforming ideas into products, using AI to inspire alternatives, solving open-ended problems, revising products, combining modalities, collaborating creatively, overcoming difficulty, and presenting ideas through digital media. In the theoretical text, however, self-efficacy is treated as the capability-belief construct rather than as a synonym for generalized confidence.

#### 3.4.3. Self-Perceived Digital Creative Functioning

Self-perceived digital creative functioning was represented by 14 contextualized items informed by digital creativity and creative-behavior research (Hoffmann et al., 2016; Shneiderman, 2007; Wu et al., 2026). The items concern perceived capability to generate, combine, revise, express, communicate, adapt, and critically improve digital outputs. Because many items use capability-oriented wording, the composite is interpreted as self-perceived functioning. It is not a frequency measure of creative behavior and does not provide externally rated evidence of novelty or usefulness.

#### 3.4.4. AI Learning Risk Awareness

AI learning risk awareness was represented by 10 study-specific items informed by work on AI anxiety, AI literacy, responsible AI use, and documented educational risks of GenAI (Chan, 2023; Y.-Y. Wang & Wang, 2022; Yan et al., 2024; Zhai et al., 2024). Items address output inaccuracy, overreliance, academic integrity, originality, bias, verification, technological pressure, possible misunderstanding, privacy, and passive learning. The resulting score is interpreted as a broad reflective risk-monitoring summary in this sample. It should not be interpreted as a fully validated general-purpose scale, as evidence that the individual risk domains are interchangeable, or as a universal reflective trait synonymous with affective AI anxiety.

### 3.5. Data Screening and Analytic Strategy

Returned questionnaires were screened before analysis for incompleteness, patterned responding, and implausibly short completion. Descriptive statistics, including means, standard deviations, skewness, and kurtosis, were computed for the composite scores. Pearson correlations provided an initial account of bivariate associations. The usable analytic sample comprised the 920 cases retained after screening.

The measurement model was evaluated with confirmatory factor analysis. Fit was assessed using chi-square divided by degrees of freedom, the comparative fit index (CFI), Tucker–Lewis index (TLI), root mean square error of approximation (RMSEA), and standardized root mean square residual (SRMR). CFI and TLI values of 0.90 or higher and RMSEA and SRMR values below 0.08 were treated as evidence of acceptable, rather than definitive, fit (Hu & Bentler, 1999; Kline, 2016). Internal consistency was examined using alpha and omega; composite reliability and average variance extracted assessed convergent evidence; HTMT and alternative-factor models assessed discriminant evidence (Fornell & Larcker, 1981; Hair et al., 2019; Henseler et al., 2015). Because statistical separation cannot by itself establish conceptual distinctness, these diagnostics were interpreted together with item wording and the construct definitions in Section 2.3.

Structural associations were estimated from observed composite scores using a regression-based second-stage conditional process model (Hayes, 2018; Preacher & Hayes, 2008; Preacher et al., 2007). Digital creative self-efficacy and AI learning risk awareness were mean-centered before their product term was formed. Indirect estimates used 5000 bootstrap resamples. Conditional indirect estimates were probed at one standard deviation below the risk-awareness mean, at the mean, and one standard deviation above the mean. Unstandardized coefficients are reported for model consistency. The conventional PROCESS term ‘index of moderated mediation’ is retained only when naming the estimator; throughout the manuscript it is interpreted as a statistical index of change in a conditional indirect association, not as evidence of causal mediation.

Demographic and digital-learning background variables were summarized descriptively but were not entered into the focal regression equations. We agree that variables such as prior GenAI experience, use frequency, self-rated digital skill, and digital-creation experience are substantively relevant and may account for heterogeneity or alternative explanations. However, the original analysis did not specify a theory-based covariate set, and this cross-sectional design does not provide a defensible causal adjustment model from which to select controls after observing the data. We therefore did not construct a post hoc battery of controls solely because the variables were available (Bernerth & Aguinis, 2016). Accordingly, all focal coefficients are explicitly labeled as unadjusted associations, and the Discussion treats these background variables as unresolved alternative correlates. This reporting choice should not be interpreted as evidence that the focal estimates are robust to those variables; covariate-adjusted sensitivity analyses based on a prespecified theoretical adjustment set remain an important task for future confirmatory work.

## 4. Results

### 4.1. Descriptive Statistics and Correlations

Table 3 reports descriptive statistics and correlations. AI learning self-efficacy was strongly and positively correlated with digital creative self-efficacy and self-perceived digital creative functioning. Digital creative self-efficacy was also strongly correlated with self-perceived digital creative functioning. AI learning risk awareness correlated positively with all three measures. This positive pattern is consistent with risk monitoring coexisting with engagement, although correlation alone cannot establish the construct’s developmental or causal role.

### 4.2. Measurement Model Evaluation

Table 4 summarizes the item counts, loading ranges, internal-consistency coefficients, composite reliability, and AVE. All reliability coefficients exceeded 0.95 and all AVE values exceeded 0.70. These values indicate very high internal consistency and strong within-sample convergent evidence. Their unusually high magnitude also raises the possibility of item redundancy, particularly for digital creative self-efficacy and self-perceived digital creative functioning; reliability is therefore not treated as proof of distinct or broad construct validity.

For measurement-diagnostic purposes, the proposed four-factor model represented AI learning self-efficacy, digital creative self-efficacy, self-perceived digital creative functioning, and the broad, study-specific AI learning risk-awareness measure as separate latent factors. The model showed acceptable fit, χ^2^/df = 5.71, CFI = 0.923, TLI = 0.919, RMSEA = 0.072, and SRMR = 0.040. Relative to the nested three-factor model that combined digital creative self-efficacy and self-perceived digital creative functioning, the four-factor model improved fit by Δχ^2^(3) = 1414.56, *p* < .001, with ΔCFI = 0.026, ΔTLI = 0.027, and ΔRMSEA = −0.011. It also fit better than the two-factor and one-factor alternatives. These comparisons provide meaningful internal-structure evidence for statistical separation in this sample, but they do not remove the semantic proximity created by overlapping capability language. The model-comparison results are summarized in Table 5.

HTMT values are shown in Table 6. All were below 0.85, but two values approached that stricter criterion: 0.842 between digital creative self-efficacy and self-perceived digital creative functioning and 0.841 between AI learning self-efficacy and digital creative self-efficacy. The combination of improved four-factor fit and near-threshold HTMT values supports cautious statistical distinction rather than strong conceptual independence. The DCE–DCF pair is especially close because both rely on self-appraisals of capability in digital creative activity; this limitation is carried forward explicitly in the Discussion.

### 4.3. Common Method Bias and Multicollinearity

All focal constructs were measured by self-report in one questionnaire, so common method variance remains a material concern. The first unrotated factor accounted for 35.03% of variance, and the proposed four-factor CFA fit substantially better than the one-factor model. These diagnostics indicate that a single undifferentiated factor did not fully reproduce the covariance matrix; they cannot rule out method-related inflation of associations. In the conditional process model, VIF values for digital creative self-efficacy, AI learning self-efficacy, AI learning risk awareness, and the interaction were 3.21, 3.40, 1.55, and 1.04, respectively, all below 5.00.

### 4.4. Structural Associations and Hypothesis Testing

**Figure 1:**
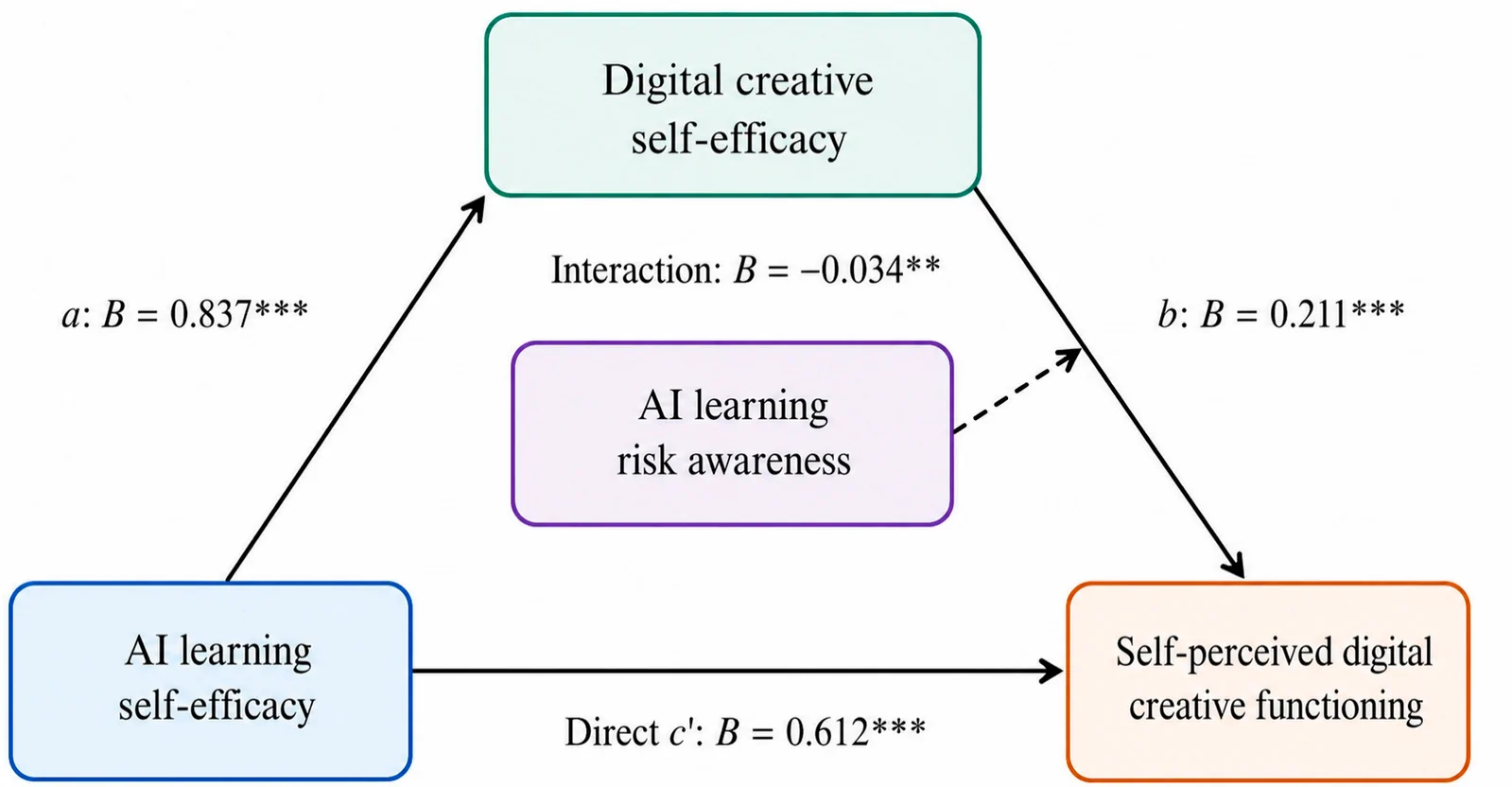
Conditional indirect-association model with estimated coefficients. Note. All displayed path estimates are unstandardized coefficients (B), matching Table 7. The direct coefficient is estimated after accounting for digital creative self-efficacy, AI learning risk awareness, and their interaction. ** *p* < .01. *** *p* < .001.

Table 7 reports the regression estimates. AI learning self-efficacy was positively associated with self-perceived digital creative functioning and with digital creative self-efficacy. In the outcome model, digital creative self-efficacy retained a positive association with self-perceived digital creative functioning, while the direct association of AI learning self-efficacy remained comparatively large and statistically significant. At mean risk awareness, the model-implied indirect estimate was 0.177, compared with a direct association of 0.612 and a total association of 0.789. The indirect component therefore represented approximately 22.4% of the total association and was partial rather than predominant. The risk-awareness interaction was negative and small. Figure 1 summarizes the conditional indirect-association model and its estimated coefficients.

### 4.5. Indirect and Conditional Indirect Associations

Using the coefficients reported in Table 7, the model-implied indirect point estimate at mean risk awareness was 0.177. Conditional point estimates decreased from 0.196 at low risk awareness to 0.157 at high risk awareness. The absolute difference was 0.039, approximately 19.9% of the low-risk estimate. These point estimates are reported descriptively in Table 8. The conventional index of moderated mediation was −0.028, 95% bootstrap CI [−0.051, −0.009]. We use this established estimator name only for identification; substantively, it indicates that the conditional indirect association decreased reliably but modestly as risk awareness increased, thereby answering RQ2.

**Figure 2:**
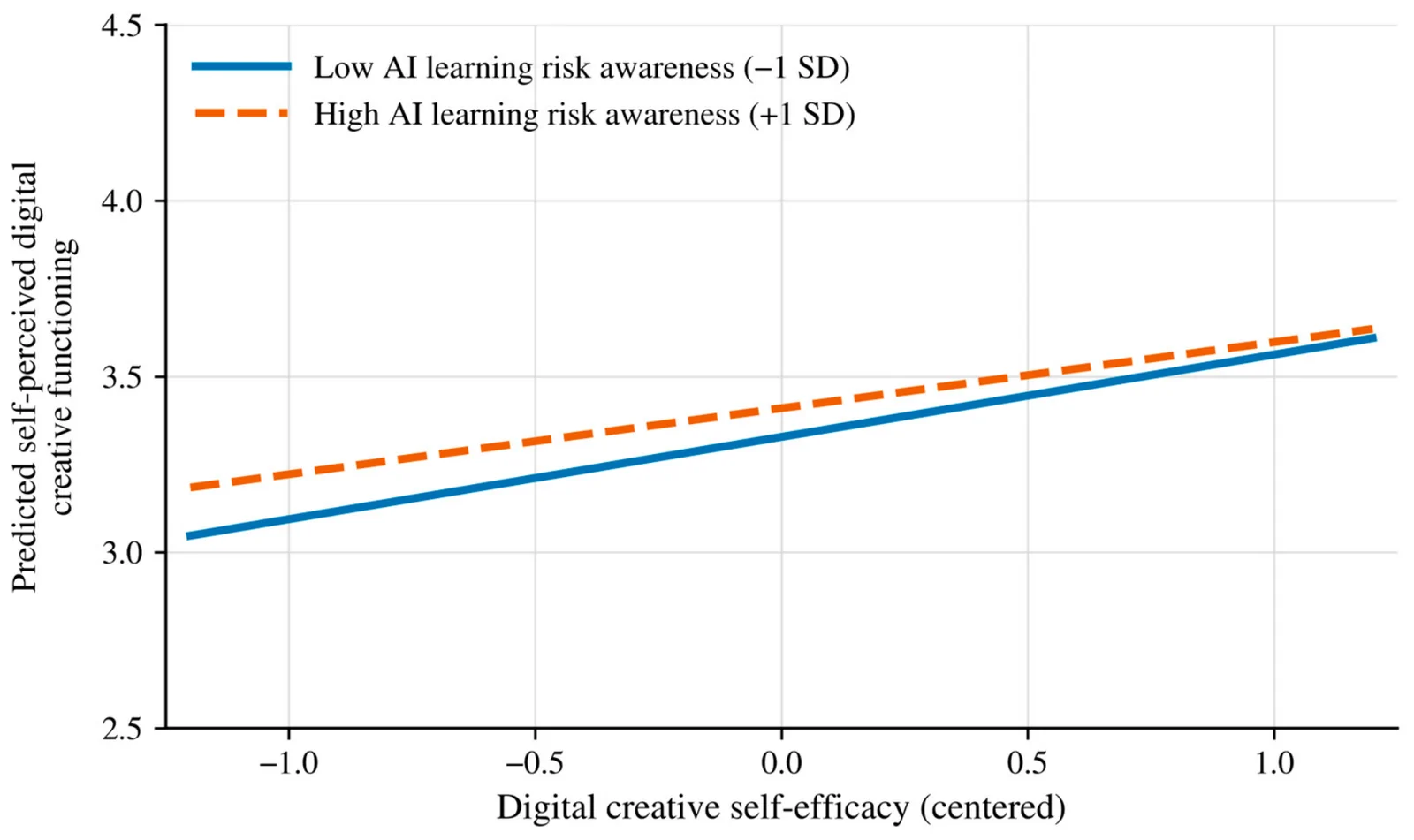
Exploratory moderation by AI learning risk awareness. Note. Lines represent predicted self-perceived digital creative functioning at low (−1 SD) and high (+1 SD) AI learning risk awareness, with AI learning self-efficacy held at its sample mean. The lines converge as digital creative self-efficacy increases, reflecting the negative interaction coefficient.

The interaction between digital creative self-efficacy and AI learning risk awareness was negative and statistically significant, B = −0.034, SE = 0.011, t = −3.09, *p* = .002. The implied unstandardized association between digital creative self-efficacy and self-perceived digital creative functioning decreased from 0.234 at low risk awareness to 0.188 at high risk awareness, indicating a modest attenuation of the positive association. The conditional indirect estimate likewise declined from 0.196 at low risk awareness to 0.157 at high risk awareness, while all three bootstrap confidence intervals excluded zero. The conventional index of moderated mediation was negative, index = −0.028, 95% bootstrap CI [−0.051, −0.009], indicating a reliable change in the conditional indirect association. Collectively, RQ1 and RQ2 identify a statistically detectable but substantively modest attenuation pattern; the positive focal association remained evident across all examined levels of risk awareness. Figure 2 illustrates this pattern.

Taken together, H1–H3 were supported as cross-sectional association hypotheses, and the positive model-implied indirect estimates were consistent with H4. RQ1 and the conventional index reported for RQ2 identified a statistically reliable but substantively small attenuation pattern. None of these results establishes temporal ordering, causal mediation, or causal moderation.

## 5. Discussion

### 5.1. Overview of Key Findings

Grounded in social cognitive theory, this study examined whether efficacy beliefs about learning with AI were associated with self-perceived digital creative functioning through a more task-specific efficacy belief. The findings are consistent with this domain-specific account. AI learning self-efficacy was strongly associated with digital creative self-efficacy, which in turn was positively associated with perceived digital creative functioning. The direct association remained comparatively large after the task-specific belief was included. At mean risk awareness, the indirect component was 0.177, compared with a direct association of 0.612, indicating a meaningful but non-dominant partial indirect association. This interpretation remains subject to the limitations of single-wave self-report data.

Risk awareness showed a dual empirical pattern. Students reporting stronger awareness also reported higher efficacy and perceived functioning, suggesting that reflective caution can coexist with engagement. The interaction was negative: the association between creative self-efficacy and perceived functioning was slightly weaker at higher risk awareness. However, the absolute change in the conditional indirect estimate was only 0.039, and the association remained positive at all probed levels. Given the large sample (N = 920), statistical detectability should not be equated with theoretical or practical importance. The moderation is therefore interpreted as a small exploratory refinement rather than a central mechanism or a strong suppressive role for risk awareness.

### 5.2. Theoretical Contributions

The first contribution is conceptual precision. Prior AI-in-education models have related self-efficacy and creativity without necessarily distinguishing general AI-learning efficacy, creative-task efficacy, and self-perceived enactment (S. Wang et al., 2023; Yang et al., 2026). The present model separates these levels of self-perception and explicitly limits the outcome claim. The pattern is consistent with efficacy specificity: broader efficacy beliefs about learning with AI are associated with perceived creative functioning partly through the narrower belief that one can execute digital creative tasks. This is a refinement of an efficacy account, not evidence that the study has isolated a causal mechanism.

The second contribution is the distinction between affective AI anxiety and risk-aware monitoring. AI-related concern is sometimes operationalized as anxiety (Y.-Y. Wang & Wang, 2022; Yang et al., 2026), whereas the GenAI literature also documents deliberate attention to inaccuracy, bias, originality, privacy, academic integrity, and overreliance (Yan et al., 2024; Zhai et al., 2024). In the present data, awareness of such risks coexisted with higher efficacy and perceived functioning while modestly attenuating one focal association. This coexistence-plus-attenuation pattern is more informative than treating all concern as avoidance. At the same time, the present indicator is broad and study-specific; the result should motivate multidimensional measurement rather than be taken as validation of a universal risk-awareness scale.

The third contribution concerns the boundary between creative self-efficacy and perceived creative functioning. The four-factor model improved substantially over a model that combined the two measures, yet HTMT was 0.842 and both measures contain capability-oriented wording. Statistical distinguishability therefore coexists with substantive conceptual and item-level overlap. Making that boundary explicit improves interpretive validity: the results concern differentiated self-perceptions within digital creative activity and do not demonstrate that students produced more original or useful work. This qualification is part of the contribution because it prevents a self-report association from being re-described as objective creativity.

A further implication is methodological restraint. The combination of very high reliability, near-threshold discriminant-validity coefficients, and a small interaction shows why multiple diagnostics should be interpreted jointly rather than treated as binary pass/fail tests. Internal-structure evidence is supportive, but criterion validity, temporal ordering, cross-sample replication, latent-structural robustness, and external creative-performance evidence remain to be established.

### 5.3. Practical Implications

The findings suggest that AI literacy programs may benefit from connecting technical competence with creative-task mastery. Prompting instruction alone may increase familiarity without necessarily strengthening efficacy for ideation, multimodal integration, iterative revision, or critical adaptation. One pedagogical option is to use scaffolded projects in which students compare alternatives, document prompt revisions, justify design decisions, and explain how their own ideas changed during the process.

The results also support treating risk awareness as something to structure rather than simply maximize. Guidance on verification, bias, privacy, authorship, overreliance, and academic-integrity expectations may reduce unproductive uncertainty while preserving critical monitoring. Because the observed attenuation was small and the design is noncausal, the findings do not show that increasing risk awareness will improve or impair creative functioning; they instead support balanced, actionable guidance rather than blanket avoidance.

Assessment should combine self-perceptions with evidence of enactment and product quality. Digital portfolios, process logs, expert ratings, and structured creative tasks can reveal whether efficacy beliefs correspond to original, useful, and personally attributable outcomes. Using multiple evidence sources would also help students understand creativity as a process of exploration, evaluation, ownership, and refinement.

### 5.4. Limitations and Future Research

The study’s principal limitation is temporal. All variables were measured at one point, so the indirect pathway does not establish that AI learning self-efficacy developed first or caused later digital creative self-efficacy or perceived creative functioning. Reverse or reciprocal associations are plausible. Longitudinal, experience-sampling, and intervention designs are needed to test change and temporal precedence.

Second, the study used a common self-report format. One-factor diagnostics indicate that a single general factor did not fully account for the data, but they cannot eliminate response consistency, social desirability, or common method inflation. Future studies should combine surveys with behavioral traces, expert-rated products, portfolio evidence, peer assessment, or creative-performance tasks.

Third, digital creative self-efficacy and self-perceived digital creative functioning remain conceptually close. The four-factor CFA and HTMT support cautious statistical separation, but the DCE–DCF HTMT value (0.842) sits near the 0.85 criterion, reliability is extremely high, and several items use overlapping capability language. Item redundancy may therefore inflate the apparent precision of the distinction. Future scale adaptation should omit or rewrite the most redundant items, reserve prospective capability-under-challenge wording for self-efficacy, and operationalize functioning with behavioral frequency, completed products, process traces, or external evaluation wherever possible.

Fourth, AI learning risk awareness is a study-specific, broad operationalization spanning accuracy, dependence, ethics, privacy, evaluation, and pressure. These domains share a risk-monitoring referent but may have different psychological antecedents and consequences. The present composite should therefore be viewed as a parsimonious empirical summary in this sample, not as proof of a universal unidimensional psychological construct. The one-factor CFA result indicates shared covariance, but it cannot by itself establish substantive unidimensionality. Future work should develop and cross-validate multidimensional measures, establish content, criterion, and incremental validity, and distinguish reflective monitoring from fear, avoidance, technostress, and institutional uncertainty.

Fifth, the present article does not claim a formal scale-development or cross-cultural-validation protocol for the study-specific item sets. The available study records do not document a separate formal expert-review/content-validation panel, pilot-testing study, or translation/back-translation validation protocol, and the internal-consistency and CFA evidence reported here cannot substitute for those procedures. We therefore deliberately restrict the validity claim to within-sample internal consistency and internal structure and provide all item wording in Appendix A for transparent inspection. Future studies should implement and report formal content validation, piloting, translation-equivalence assessment where relevant, criterion validation, and independent cross-sample replication before using the item sets as general-purpose instruments.

Sixth, sample composition limits generalization. Most participants were first-year students from regular undergraduate institutions, and STEM students were overrepresented. Students were drawn from higher education settings in China, where policy, assessment, and platform use may differ from other contexts. Replication should use institutionally diverse samples and account for clustering within courses and universities when those identifiers are available.

Finally, the focal regression coefficients are unadjusted associations. Variables reported descriptively in Table 1 and Table 2—including prior GenAI experience, frequency of AI use, self-rated digital skill, and digital-creation/project experience—could account for heterogeneity or provide alternative explanations for the observed associations. Because the original analysis did not specify a theory-based covariate set, we did not retrospectively select a large control set solely from variable availability. Consequently, the present estimates should not be interpreted as demonstrating robustness to these background variables. Future confirmatory studies should preregister theoretically justified covariates and report adjusted sensitivity analyses alongside unadjusted focal estimates. The moderation itself was statistically reliable but small, further reinforcing the need to separate statistical detection from substantive importance.

## 6. Conclusions

AI learning self-efficacy was positively associated with self-perceived digital creative functioning, with a smaller statistical indirect component through digital creative self-efficacy. At mean risk awareness, the model-implied indirect estimate was 0.177, compared with a direct association of 0.612, supporting a partial rather than predominant indirect-association account. AI learning risk awareness coexisted with efficacy and functioning while modestly attenuating the focal association; this statistically detectable pattern was substantively small. The findings support a domain-specific efficacy interpretation and careful risk monitoring as topics for further study, but they do not establish that changing either construct would cause changes in creative functioning. These single-wave self-report results concern perceived functioning and do not establish causal order or objectively rated creativity.

## Figures and Tables

**Table 1:** Demographic characteristics of participants (*N* = 920).

Variable	Category	*n*	%
**Gender**	Male	357	38.8
	Female	563	61.2
**Academic year**	First-year undergraduate	543	59.0
	Second-year undergraduate	267	29.0
	Third-year undergraduate	107	11.6
	Fourth-year undergraduate	3	0.3
**Discipline**	STEM	521	56.6
	Humanities and social sciences	107	11.6
	Economics, management, and law	52	5.7
	Arts and sports	44	4.8
	Other	196	21.3
**University type**	Double First-Class/key university	10	1.1
	Regular undergraduate university	754	82.0
	Higher vocational college	151	16.4
	Other	5	0.5
**Academic performance**	Top 10%	100	10.9
	Top 11–25%	170	18.5
	Top 26–50%	291	31.6
	Lower 50%	118	12.8
	Not sure	241	26.2

Note. Percentages may not sum to 100 because of rounding.

**Table 2:** Digital-learning and GenAI-use background characteristics (*N* = 920).

Variable	Category	*n*	%
**Weekly digital-learning time**	Less than 5 h	416	45.2
	5–10 h	303	32.9
	11–15 h	109	11.8
	16–20 h	39	4.2
	More than 20 h	53	5.8
**GenAI-use duration**	Never used	24	2.6
	Less than 3 months	63	6.8
	3–6 months	170	18.5
	6–12 months	188	20.4
	More than 1 year	475	51.6
**Weekly GenAI learning frequency**	Never	29	3.2
	1–2 times	159	17.3
	3–4 times	290	31.5
	5–6 times	131	14.2
	Almost every day	311	33.8
**Digital-creation/project experience**	Never	297	32.3
	Occasionally	378	41.1
	Once/one course	154	16.7
	2–3 times/courses	62	6.7
	Frequently	29	3.2
**Self-rated digital skill**	Very low	70	7.6
	Low	152	16.5
	Average	600	65.2
	High	79	8.6
	Very high	19	2.1

Note. Percentages may not sum to 100 because of rounding. GenAI tools included tools such as ChatGPT, Kimi, Tongyi, Doubao, and Wenxin Yiyan.

**Table 3:** Descriptive statistics and correlations among focal variables.

Variable	M	SD	Skewness	Kurtosis	1	2	3	4
1. AI learning self-efficacy	3.53	0.71	−0.16	1.30	—			
2. Digital creative self-efficacy	3.34	0.72	0.17	1.37	0.822 ***	—		
3. Self-perceived digital creative functioning	3.36	0.72	0.05	1.62	0.783 ***	0.712 ***	—	
4. AI learning risk awareness	3.44	0.68	−0.05	1.54	0.580 ***	0.541 ***	0.542 ***	—

Note. Pearson correlation coefficients are reported. *** *p* < .001.

**Table 4:** Operationalization sources, reliability, and convergent evidence.

Construct	Source	Items	Loading Range	α	ω	CR	AVE
AI learning self-efficacy (AILE)	AI self-efficacy and AI literacy literature	10	0.895–0.926	0.977	0.980	0.980	0.831
Digital creative self-efficacy (DCE)	Creative self-efficacy literature	10	0.906–0.926	0.979	0.981	0.981	0.840
Self-perceived digital creative functioning (DCF)	Digital creativity and creative-behavior literature	14	0.824–0.921	0.979	0.981	0.981	0.784
AI learning risk awareness (AILRA)	AI anxiety, AI literacy, and responsible-AI literature	10	0.799–0.870	0.955	0.961	0.961	0.714

Note. α = Cronbach’s alpha; ω = McDonald’s omega; CR = composite reliability; AVE = average variance extracted. Loading ranges are standardized item-factor loading estimates based on the present sample.

**Table 5:** Confirmatory factor analysis model comparison.

Model	χ^2^	df	χ^2^/df	CFI	TLI	RMSEA	SRMR
Four-factor model	5116.93	896	5.71	0.923	0.919	0.072	0.040
Three-factor model (DCE and DCF combined)	6531.49	899	7.27	0.897	0.892	0.083	0.043
Two-factor model (positive learning beliefs combined)	11,183.73	901	12.42	0.812	0.803	0.111	0.062
One-factor model	16,588.96	902	18.39	0.713	0.699	0.138	0.108

Note. The proposed four-factor model treated AI learning self-efficacy, digital creative self-efficacy, self-perceived digital creative functioning, and AI learning risk awareness as separate factors. In the three-factor alternative, digital creative self-efficacy and self-perceived digital creative functioning were combined. CFI = comparative fit index; TLI = Tucker–Lewis index; RMSEA = root mean square error of approximation; SRMR = standardized root mean square residual.

**Table 6:** Discriminant validity based on HTMT.

Construct	AILE	DCE	DCF	AILRA
AILE	1.000			
DCE	0.841	1.000		
DCF	0.801	0.842	1.000	
AILRA	0.602	0.560	0.561	1.000

Note. AILE = AI learning self-efficacy; DCE = digital creative self-efficacy; DCF = self-perceived digital creative functioning; AILRA = AI learning risk awareness. Values below 0.85 were treated as evidence of cautious statistical distinction. Near-threshold values should be interpreted together with item content and the alternative CFA models; they are not taken as proof of conceptual independence.

**Table 7:** Regression coefficients for the conditional indirect-association model.

Path	B	SE	t	*p*
AI learning self-efficacy → Self-perceived digital creative functioning (total association)	0.789	0.021	38.17	<.001
AI learning self-efficacy → Digital creative self-efficacy	0.837	0.019	43.69	<.001
Digital creative self-efficacy → Self-perceived digital creative functioning	0.211	0.023	9.17	<.001
AI learning self-efficacy → Self-perceived digital creative functioning (direct association)	0.612	0.024	25.50	<.001
AI learning risk awareness → Self-perceived digital creative functioning	0.060	0.017	3.46	<.001
Digital creative self-efficacy × AI learning risk awareness → Self-perceived digital creative functioning	−0.034	0.011	−3.09	.002

Note. Unstandardized, unadjusted coefficients are reported. Model 1 estimates the total association between AI learning self-efficacy and self-perceived digital creative functioning. Model 2 predicts the observed composite score for digital creative self-efficacy. Model 3 predicts the observed composite score for self-perceived digital creative functioning and includes the second-stage interaction. Digital creative self-efficacy and AI learning risk awareness were mean-centered before constructing the product term. Bootstrap resamples = 5000. Coefficients are cross-sectional associations; demographic and background variables were not included as automatic controls.

**Table 8:** Conditional indirect association estimates and conventional index of moderated mediation.

AILRA Level/Statistic	Centered Value	Estimate	95% CI LL	95% CI UL
Low AILRA (−1 SD)	−0.68	0.196	0.156	0.237
Mean AILRA	0.00	0.177	0.138	0.215
High AILRA (+1 SD)	0.68	0.157	0.117	0.198
Index of moderated mediation	—	−0.028	−0.051	−0.009

Note. Unstandardized conditional indirect association estimates are reported. AI learning risk awareness (AILRA) was mean-centered, with low, mean, and high levels corresponding to one standard deviation below the mean (−0.68), the mean (0.00), and one standard deviation above the mean (0.68), respectively. Point estimates and 95% bootstrap confidence intervals were obtained from 5000 bootstrap resamples. The conventional index of moderated mediation is reported only as the established statistical label for the linear change in the conditional indirect association associated with a one-unit increase in AILRA; it is not interpreted causally. CI = confidence interval; LL = lower limit; UL = upper limit; AILRA = AI learning risk awareness.

## Data Availability

The data are not deposited in an unrestricted public repository because release is subject to institutional ethics requirements and participant-privacy safeguards. A de-identified dataset and analysis information may be made available to qualified researchers on reasonable request, subject to institutional approval.

## Appendix A. Scale Item Wording and Construct Distinction

The complete wording is provided to make the study-specific operationalizations transparent. AILE and DCE assess efficacy-oriented capability beliefs. DCF is more enactment-oriented but still contains capability wording and is therefore interpreted as self-perceived digital creative functioning rather than objectively rated creativity. AILRA is a broad, study-specific risk-monitoring measure in this sample rather than an established unidimensional construct. The full wording is reported so that readers can evaluate the conceptual proximity and possible item redundancy discussed in the manuscript.

**Table A1:** Study-specific constructs and item wording.

Item	Construct	Wording
AILE1	AI learning self-efficacy	I can learn to use new generative AI tools effectively for my coursework.
AILE2	AI learning self-efficacy	I can write prompts that help AI tools generate useful learning materials.
AILE3	AI learning self-efficacy	I can evaluate whether AI-generated content is accurate and relevant.
AILE4	AI learning self-efficacy	I can revise AI-generated content into my own learning product.
AILE5	AI learning self-efficacy	I can use AI tools to organize complex learning materials.
AILE6	AI learning self-efficacy	I can solve problems that arise when using AI tools for learning.
AILE7	AI learning self-efficacy	I can compare different AI-generated answers and select the most appropriate one.
AILE8	AI learning self-efficacy	I can use AI tools to support independent learning.
AILE9	AI learning self-efficacy	I can avoid inappropriate use of AI in academic tasks.
AILE10	AI learning self-efficacy	I can integrate AI suggestions with my own understanding.
DCE1	Digital creative self-efficacy	I am confident that I can generate original ideas for digital projects.
DCE2	Digital creative self-efficacy	I am confident that I can use digital tools to develop creative solutions.
DCE3	Digital creative self-efficacy	I am confident that I can transform a rough idea into a digital product.
DCE4	Digital creative self-efficacy	I am confident that I can use AI tools to inspire creative alternatives.
DCE5	Digital creative self-efficacy	I am confident that I can solve open-ended digital problems creatively.
DCE6	Digital creative self-efficacy	I am confident that I can revise digital products to make them more creative.
DCE7	Digital creative self-efficacy	I am confident that I can combine text, image, audio, or video creatively.
DCE8	Digital creative self-efficacy	I am confident that I can contribute creative ideas in digital collaboration.
DCE9	Digital creative self-efficacy	I am confident that I can overcome difficulties in digital creative tasks.
DCE10	Digital creative self-efficacy	I am confident that I can present creative ideas through digital media.
DC1	Self-perceived digital creative functioning	I often generate novel ideas when completing digital tasks.
DC2	Self-perceived digital creative functioning	I can combine different digital materials in original ways.
DC3	Self-perceived digital creative functioning	I can create digital products that communicate ideas clearly.
DC4	Self-perceived digital creative functioning	I can revise digital works to improve originality and usefulness.
DC5	Self-perceived digital creative functioning	I can use digital tools to express ideas in multiple formats.
DC6	Self-perceived digital creative functioning	I can create digital outputs that differ from common solutions.
DC7	Self-perceived digital creative functioning	I can integrate text, images, audio, and video into coherent products.
DC8	Self-perceived digital creative functioning	I can identify new possibilities when using digital technologies.
DC9	Self-perceived digital creative functioning	I can design digital products that are useful to others.
DC10	Self-perceived digital creative functioning	I can present complex ideas through appropriate digital channels.
DC11	Self-perceived digital creative functioning	I can adapt existing digital materials for new purposes.
DC12	Self-perceived digital creative functioning	I can use AI-assisted suggestions to improve creative products critically.
DC13	Self-perceived digital creative functioning	I can produce digital work that reflects my own ideas.
DC14	Self-perceived digital creative functioning	I can evaluate and refine digital products based on creative goals.
AILRA1	AI learning risk awareness	I pay attention to the possibility that AI-generated content may be inaccurate.
AILRA2	AI learning risk awareness	I am aware that overreliance on AI may weaken my independent thinking.
AILRA3	AI learning risk awareness	I consider academic-integrity risks when using AI tools for learning.
AILRA4	AI learning risk awareness	I think carefully about whether AI-assisted work is sufficiently original.
AILRA5	AI learning risk awareness	I am aware that AI outputs may contain bias or misleading information.
AILRA6	AI learning risk awareness	I feel the need to verify AI-generated information with other sources.
AILRA7	AI learning risk awareness	I am aware that rapid changes in AI tools may create learning pressure.
AILRA8	AI learning risk awareness	I consider whether teachers or peers may misunderstand AI-assisted work.
AILRA9	AI learning risk awareness	I pay attention to privacy or data risks when using AI tools.
AILRA10	AI learning risk awareness	I reflect on whether AI use makes my learning too passive.

Note. Items use a five-point response format from 1 = strongly disagree to 5 = strongly agree. AILE = AI learning self-efficacy; DCE = digital creative self-efficacy; DCF = self-perceived digital creative functioning; AILRA = AI learning risk awareness. The measures are study-specific operationalizations informed by the cited studies.
